# Targeted HIV-1 Latency Reversal Using CRISPR/Cas9-Derived Transcriptional Activator Systems

**DOI:** 10.1371/journal.pone.0158294

**Published:** 2016-06-24

**Authors:** Julia K. Bialek, Gábor A. Dunay, Maike Voges, Carola Schäfer, Michael Spohn, Rolf Stucka, Joachim Hauber, Ulrike C. Lange

**Affiliations:** 1 Heinrich Pette Institute – Leibniz Institute for Experimental Virology, Hamburg, Germany; 2 Department of Anesthesiology, University Medical Center Hamburg-Eppendorf, Hamburg, Germany; 3 Friedrich-Baur-Institute, Department of Neurology, Ludwig Maximilian University Munich, Munich, Germany; 4 German Center for Infection Research (DZIF), partner site, Hamburg, Germany; George Mason University, UNITED STATES

## Abstract

CRISPR/Cas9 technology is currently considered the most advanced tool for targeted genome engineering. Its sequence-dependent specificity has been explored for locus-directed transcriptional modulation. Such modulation, in particular transcriptional activation, has been proposed as key approach to overcome silencing of dormant HIV provirus in latently infected cellular reservoirs. Currently available agents for provirus activation, so-called latency reversing agents (LRAs), act indirectly through cellular pathways to induce viral transcription. However, their clinical performance remains suboptimal, possibly because reservoirs have diverse cellular identities and/or proviral DNA is intractable to the induced pathways. We have explored two CRISPR/Cas9-derived activator systems as targeted approaches to induce dormant HIV-1 proviral DNA. These systems recruit multiple transcriptional activation domains to the HIV 5’ long terminal repeat (LTR), for which we have identified an optimal target region within the LTR U3 sequence. Using this target region, we demonstrate transcriptional activation of proviral genomes via the synergistic activation mediator complex in various *in culture* model systems for HIV latency. Observed levels of induction are comparable or indeed higher than treatment with established LRAs. Importantly, activation is complete, leading to production of infective viral particles. Our data demonstrate that CRISPR/Cas9-derived technologies can be applied to counteract HIV latency and may therefore represent promising novel approaches in the quest for HIV elimination.

## Introduction

HIV latency constitutes the chief barrier to curative therapy in HIV-infected subjects [1]. It designates a state of viral dormancy established in resting CD4+ T cells and other long-lived immune cell populations upon hours after viral infection. Dormancy is characterized by transcriptional silencing of the integrated proviral genome through various mechanisms [2–4]. Although a rare occurrence with an estimated frequency of 1 to 60 cells per million CD4+ T cells, latently-infected HIV reservoirs persist for years in infected individuals, evading immune surveillance or antiretroviral therapy [5,6]. While thought to be largely harmless in their latent state, reservoir cells retain the capacity to spontaneously activate the provirus and therefore persistently spread virus. Current strategies for HIV cure focus primarily on eradicating latent reservoirs through inducing proviruses to form viral particles, and subsequent clearing of infected cells by cytopathic viral or immune surveillance mechanisms [1,7].

Several classes of latency reversing agents (LRAs) for pharmacological induction of proviral transcription have been tested in *ex vivo* latency models or clinical trials. These include protein kinase C agonists (e.g. phorbol esters, Prostratin, Bryostatin); histone deacetylase inhibitors (HDACis, e.g. Vorinostat/SAHA, Romidepsin), and bromodomain and extraterminal bromodomain inhibitors (BETis, e.g. JQ-1) [8–12]. These agents have been found to activate transcription by inducing T cell activation through signaling pathways, modulating epigenetic states, or regulating transcriptional elongation [13]. However clinical performance of current LRAs remains suboptimal. So far no trials have shown significant reduction in latent reservoirs upon LRA treatment combined with antiretroviral therapy [1,14]. Inefficient clearance of activated reservoir cells might partly explain these findings. However, several lines of evidence also indicate that transcriptional activation of latent HIV genomes by currently available LRAs is insufficient. First, a subset of latent proviruses in CD4+ T cells of HIV infected individuals was found to be intractable to *ex vivo* stimulation by LRAs [6]. Second, for latent cells of non-CD4+ identity, such as myeloid-derived microglial cells or macrophages, the activity of some LRAs has been reported as poor [9]. In line with these observations, HIV latency was described to be predominantly viral-encoded and cellular targets of current LRAs might play a secondary role in transcriptional regulation [15].

Approaches that promote transcriptional activation of proviral DNA independently of the cellular state could circumvent these drawbacks. One possible strategy is to directly target transcriptional activators to HIV proviral LTR sequences. Indeed, both zinc finger protein-derived modulators and transcriptional activator-like effector (TALE) proteins designed to be specific for HIV LTR mediate activation in latency models [16–18]. Directed transcriptional activation can also be achieved using CRISPR/Cas9-derived technologies (CRISPR: clustered regularly interspaced palindromic repeats; Cas9: CRISPR-associated protein 9) [19–24]. As RNA-dependent DNA-binding effectors, these technologies have the substantial advantage that targeting specificity is programmable simply through choosing appropriate guide RNA (gRNA) sequences.

Here, we investigated whether CRISPR/Cas9-derived activation systems can be applied to reverse HIV latency. We explored two systems: the SunTag and synergistic activation mediator (SAM) systems [25,26]. Both technologies rely on recruiting multiple transcriptional activation domains to a DNA target using sequence-complementary gRNA and enzymatically inactive *Streptococcus pyogenes* (Sp) Cas9 nuclease-derived proteins. We identified an optimal target region in the U3 sequence of the HIV 5’LTR, and demonstrate that recruitment of SAM in particular induced robust transcriptional activation of HIV genomes in various *in vitro* latency models. Importantly, levels of activation were comparable or indeed superior to those observed after exposure to established LRAs. Reversal of latency was apparently complete, with induction not only at the RNA and protein level, but also generating infectious particles. Our data present proof of concept that CRIPSR/Cas9-derived activation systems meet all the prerequisites for novel tools to complement current strategies for HIV latency reversal.

## Results

### Identifying an optimal target region for CRISPR/Cas9-derived activation systems in the HIV 5’LTR

**Fig 1:**
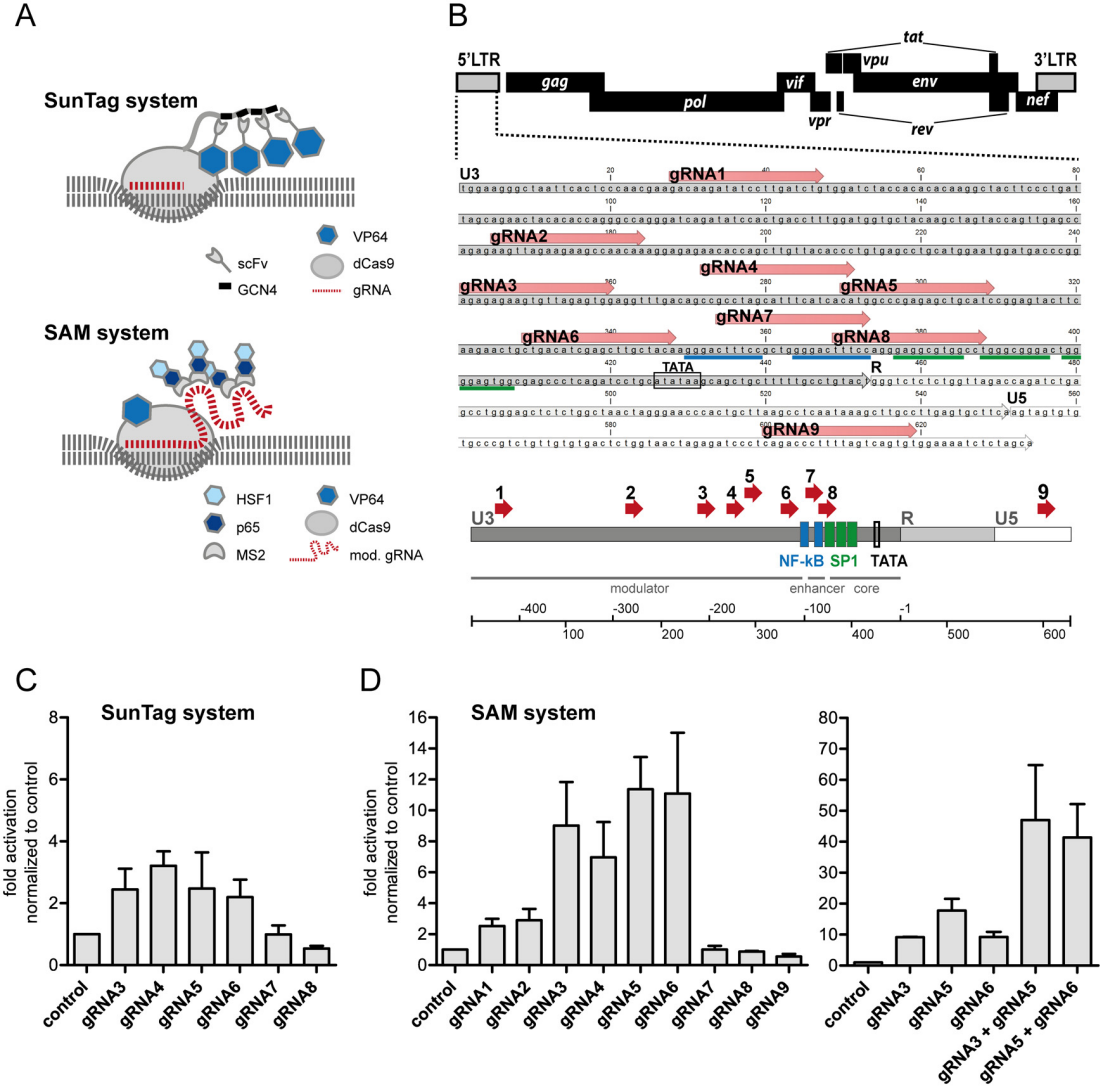
Identifying an optimal target region for SunTag and synergistic activation mediator (SAM) systems in the HIV 5‘LTR. (**A**) Scheme of SunTag and SAM activation systems. In both systems binding of a guide RNA (gRNA) to a specific target DNA sequence results in recruitment of multiple activator domains (VP64, HSF1, p65; see text for details). (**B**) HIV 5’LTR (HXB2) sequence with complimentary gRNAs1-9. (**C, D**) The effect of SunTag (**C**) and SAM (**D**) systems on LTR activation was tested by transient expression of gRNAs and system components in TZM-bl cells carrying a luciferase reporter under HIV LTR control. Activation levels were measured 48 h post transfection using a luciferase assay on whole cell lysates. Activation is shown as fold increase (light units/transfected cell) over the negative control (no gRNA expression). Shown are results of three (C; D, left panel) and two (D, right panel) independent experiments.

We explored two CRISPR/Cas9-derived activator systems for HIV transcriptional induction (Fig 1A). In the SunTag system synthetic transcriptional activator VP64 (derived from four copies of the herpes virus transcriptional activation domain VP16) is fused to single chain variable fragment (scFv) antibodies with binding specificity for peptides derived from the general control protein 4 (GCN4) [26]. Co-expression of these antibodies together with dCas9 (Cas9 nuclease-derived enzymatically inactive protein) fused to GCN4-containing peptide tags (SunTag) and gRNAs, leads to spatial recruitment of multiple VP64 domains to the gRNA-complementary target sites. The SAM (synergistic activation mediator) system [25] comprises three components: dCas9-VP64 fusion protein, modified gRNA (mod. gRNA) containing aptamers bound by MS2 bacteriophage coat protein, and MS2 proteins fused to NF-κB *trans*-activating subunit p65 and heat-shock factor 1 (HSF1) activation domain. Co-expression of these components establishes a multi activation domain-containing SAM complex mediating transcriptional activation at the gRNA-determined target site.

To identify an optimal target region for functionally recruiting SunTag and SAM systems to HIV proviral DNA, we first designed several gRNAs (gRNA1-9) specific for HIV 5’LTR. We focused in particular on a region within the U3 sequence (gRNA3-8), that is known to harbor a number of transcription factor binding sites and has recently been successfully exploited for TALE-mediated activation (Fig 1B) [16]. NGG SpCas9 protospacer adjacent motif (PAM) sites determined the gRNA sequence choice. Components of the SunTag (Fig 1C) and SAM (Fig 1D) systems together with single gRNAs were transiently expressed in HeLa cell-derived clonal TZM-bl cells carrying an HIV LTR-dependent luciferase reporter [27]. SunTag and SAM components expressed without any gRNA served as negative controls. For both systems we observed strong activation of reporter gene expression when targeting with gRNA3-6. While SunTag-mediated activation was generally lower, with a maximum of three-fold activation over controls (gRNA4), SAM-mediated activation reached levels of up to 11-fold over controls (gRNA5 and gRNA6; Fig 1C and 1D). Notably, induction of luciferase transcription was highly specific to the recruiting gRNA, with gRNA7-9 failing to induce reporter gene expression above background levels.

These results indicate an optimal target region spanning gRNA3-6 located 230 bp to 106 bp upstream of the transcriptional start site (TSS) for directed transcriptional activation of HIV 5’LTR using the SunTag and SAM systems. In particular the SAM system showed strong effects on reporter gene transcription in TZM-bl cells. Notably, these effects are potentiated by co-expression of two gRNAs within the optimal target region, leading to synergistic activation conceivably through recruitment of multiple SAM complexes (Fig 1D). Moreover, activation achieved through SAM system recruitment was also superior to reporter induction by dCas9-VP64 alone (S1 Fig).

### SAM-mediated reversal of HIV latency in T cell-derived model systems

Next we investigated whether CRISPR/Cas9-derived activation systems could be used to reverse transcriptional latency of HIV genomes, a key aspect in current purging approaches to eliminate HIV reservoirs. We focused on the SAM system using gRNA5 for optimal targeting, based on its superior activating effects seen in TZM-bl cells. We explored two T cell-derived latency models, the widely used JLat model [28] and a newly established dual-color HIV reporter model system [29].

**Fig 2:**
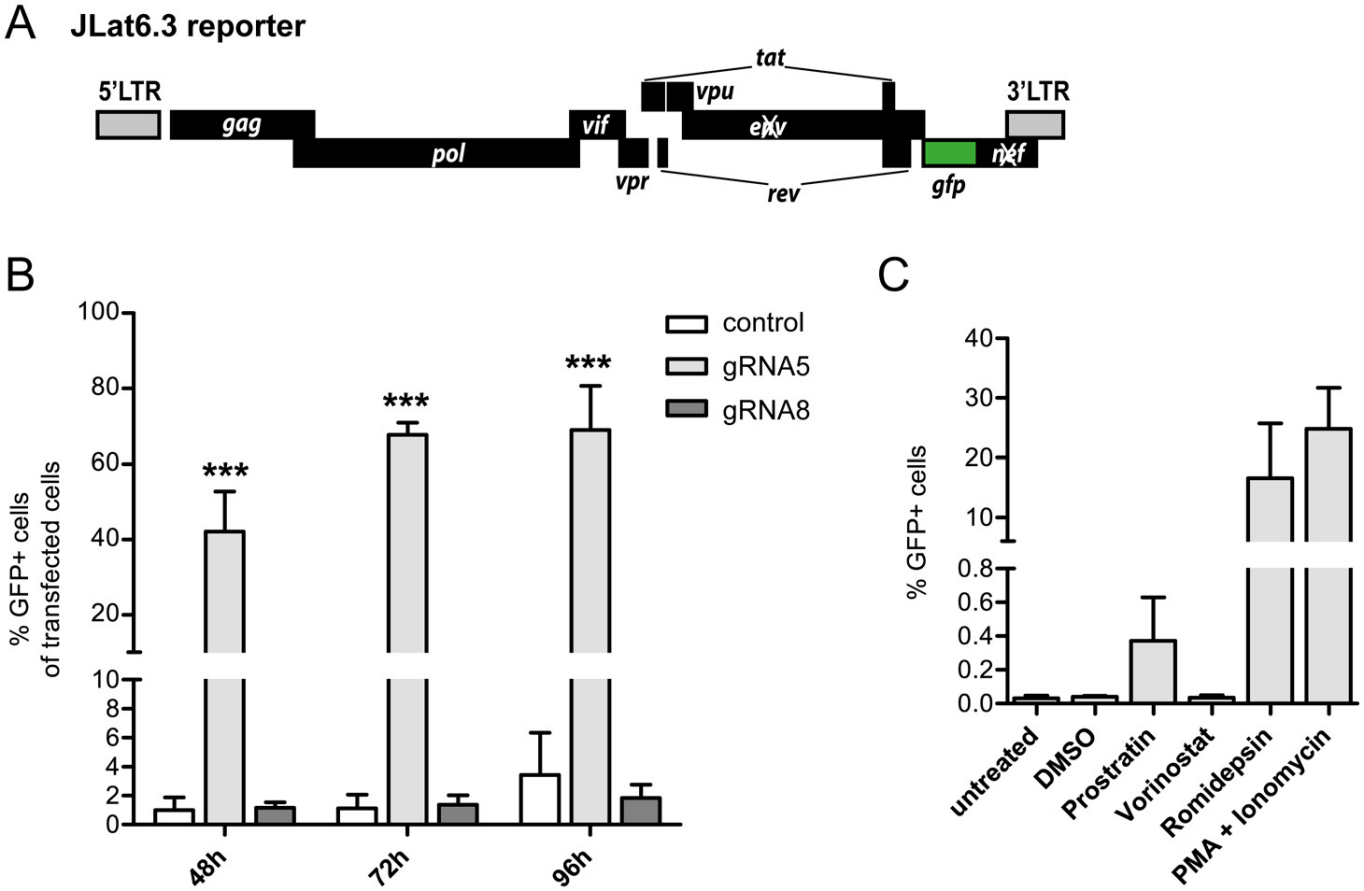
SAM-mediated latency reversal in the JLat model. (**A**) Scheme of the replication-incompetent HIV reporter virus present in Jlat6.3 cells. Transcriptional activation results in LTR-dependent eGFP expression. (**B**) Components of the SAM system together with gRNA5, gRNA8 or no gRNA (control) were transiently expressed in JLat6.3 cells and proviral activation was measured by flow cytometry at 48, 72, and 96 h post transfection. Two-way ANOVA was used for statistical evaluation (in relation to control); *** signifies p<0.001. (**C**) Proviral activation levels in JLat6.3 cells after 24 h exposure to different latency reversing agents as measured by flow cytometry. Shown are results of three independent experiments (B, C).

Jurkat-derived clonal JLat6.3 cells carry a replication-incompetent HIV-derived GFP reporter (Fig 2A), which is silenced in untreated cells, resembling a state of viral latency. To test SAM-mediated activation potential, JLat6.3 clones were transiently co-transfected with components of the SAM system plus optimal target gRNA5, gRNA8 (complementary to a negative target sequence, see Fig 1), or no gRNA (control). GFP reporter gene expression levels were measured by flow cytometry at 48, 72 and 96 h post transfection. Importantly, expression of SAM components in control cells did not result in significant activation of reporter transcription, indicating a robust silenced state that remains unperturbed by transient transfection. However the HIV reporter in JLat6.3 cells was strongly activated upon expression of the SAM complex targeted through the optimal gRNA5. Induced cells reached levels of 42% as early as 48 h post transfection and further increased to around 69% GFP-positive cells at 96 h (Fig 2B). Moreover, induction was highly dependent on the gRNA5 sequence, with no significant activation seen with gRNA8 (Fig 2B).

We then compared SAM-mediated reporter induction levels to those achieved by treating JLat6.3 cells with established LRAs used at clinically relevant concentrations as previously reported [30]. The HDAC inhibitor Vorinostat and protein kinase C agonist Prostratin only minimally induced the GFP reporter after 24 h of exposure (Fig 2C). Treatment with HDAC inhibitor Romidepsin or phorbol 12-myristate 13-acetate (PMA) and Ionomycin, a combination used for maximal *ex vivo* stimulation of HIV expression in viral outgrowth assays, led to induction levels of around 17% and 25% in the JLat model, respectively (Fig 2C). Exposure to higher LRA concentrations only moderately increased induction rates and often resulted in reduced cell viability (S2 Fig).

Taken together these results demonstrate strong SAM-mediated activation of latent HIV-reporter in JLat6.3 cells, with induction levels superior to those achieved using established LRAs.

**Fig 3:**
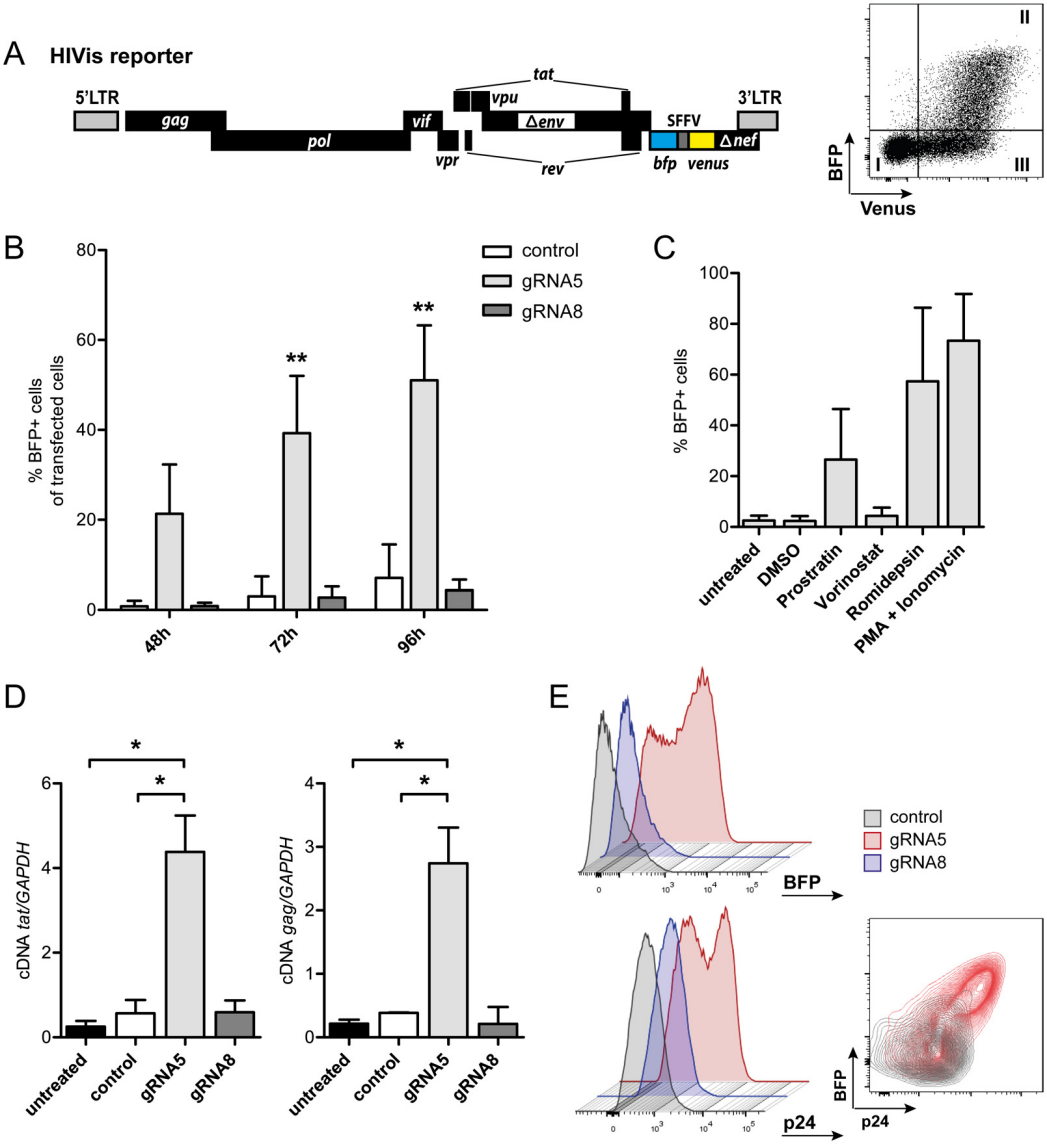
Latency reversal using SAM in the HIVis model system. (**A**) Scheme of replication incompetent HIVis latency reporter virus (left panel). Transcriptional activation results in LTR-dependent BFP expression, while the SFFV promoter drives constitutive expression of Venus fluorescent protein. Upon transduction of Jurkat cells with HIVis reporter, cells with active LTR transcription (II: BFP+, Venus+) can be distinguished from cell populations with a silenced LTR (III: BFP-, Venus+) or non-transduced cells (I: BFP-, Venus-) by flow cytometry (right panel). (**B**) Components of the SAM system together with gRNA5, gRNA8 or no gRNA (control) were transiently expressed in clonal HIVis cells (HIVisB2). Proviral activation was monitored by flow cytometry at 48, 72, and 96 h post transfection. Two-way ANOVA was used for statistical evaluation (in relation to control); ** signifies p<0.01. (**C**) Proviral activation levels in HIVisB2 cells after 24 h exposure to different latency reversing agents. (**D**) Levels of cell-associated *tat* and *gag* RNA in HIVisB2 cells as measured by qRT-PCR at 72 h post transfection with SAM system components plus gRNA5, gRNA8 or no gRNA (control). One-way ANOVA was used for statistical evaluation; * signifies p<0.05. Shown are results of three (B, C) and two (D) independent experiments. (**E**) Number of HIVisB2 cells showing BFP expression (upper panel) and cell-associated p24 expression (lower panel) at 72 h post transfection with SAM system components and gRNA5, gRNA8 or no gRNA (control). Insert shows a flow cytometry contour plot of p24 and BFP co-expression in control (grey) and SAM/gRNA5 transfected (red) cells.

Different *in vitro* models of HIV latency have shown variable responses to LRAs [31]. To test whether SAM-mediated latency reversal also demonstrates variable effects, we used a second, newly established latency system. This system, called HIVis (*HIV vis*ible), comprises an HIV-1 reporter variant [29,32] with a dual fluorescent marker cassette inserted into the *nef* locus within an HIV-1-derived replication-incompetent vector backbone. The marker cassette encodes blue fluorescent protein (BFP) under HIV LTR control and Venus-fluorescent protein under the control of constitutive spleen focus-forming virus (SFFV) promoter (Fig 3A, left). Upon transduction with HIVis reporter, flow cytometry analysis can readily distinguish between non-transduced cells and transduced cell populations with different states of HIVis reporter transcriptional activity, thereby enabling visualization of productive versus latent infection (Fig 3A, right panel). Notably, latent HIVis reporter is responsive to LRA exposure, which induces viral gene expression at RNA and protein level (S3 Fig).

We generated a clonal Jurkat cell-derived HIVis cell line (HIVisB2), carrying a single HIVis reporter integrated at the *nup188* locus in a latent state. LTR-dependent transcription, as measured by BFP expression, is minimal or absent in HIVisB2 cells (<3% of the total cell population), but can be induced by exposure to LRAs (S1 Fig). This HIVisB2 model was then used to test SAM-dependent LTR activation (Fig 3B). As observed with JLat6.3 clones, transient expression of SAM together with optimal gRNA5 induced strong expression of the latent HIV reporter: levels of activated cells were 21% after 48 h, reaching 51% at 96 hours. Again, this induction was highly specific for recruitment to the optimal target region, with no LTR transcription seen in control cells or cells co-expressing gRNA8 (Fig 3B). In comparison, treating HIVisB2 cells with clinically relevant LRAs led to induction levels of 4% (Vorinostat), 27% (Prostratin) and 57% (Romidepsin). Stronger induction could be achieved by using increased LRA concentrations, this however lead to marked reduction in viability for the HDAC inhibitors (S2 Fig). Stimulation with the most potent *ex vivo* agents PMA and Ionomycin led to robust activation levels of around 73% (Fig 3C).

Importantly, we confirmed that expression of the *bfp* marker in SAM-activated HIVisB2 cells correlated with up-regulation of cell-associated *tat* and *gag* RNA (Fig 3D). Furthermore, these cells also expressed cell-associated p24 protein, indicating that SAM-mediated activation via gRNA5 recruitment reversed HIV silencing in HIVisB2 cells (Fig 3E).

Taken together, these data demonstrate that SAM effectively activates transcription of latent HIV genomes at high levels across different *in vitro* model systems.

### Exposure to SAM converts latent state into productive viral life cycle

**Fig 4:**
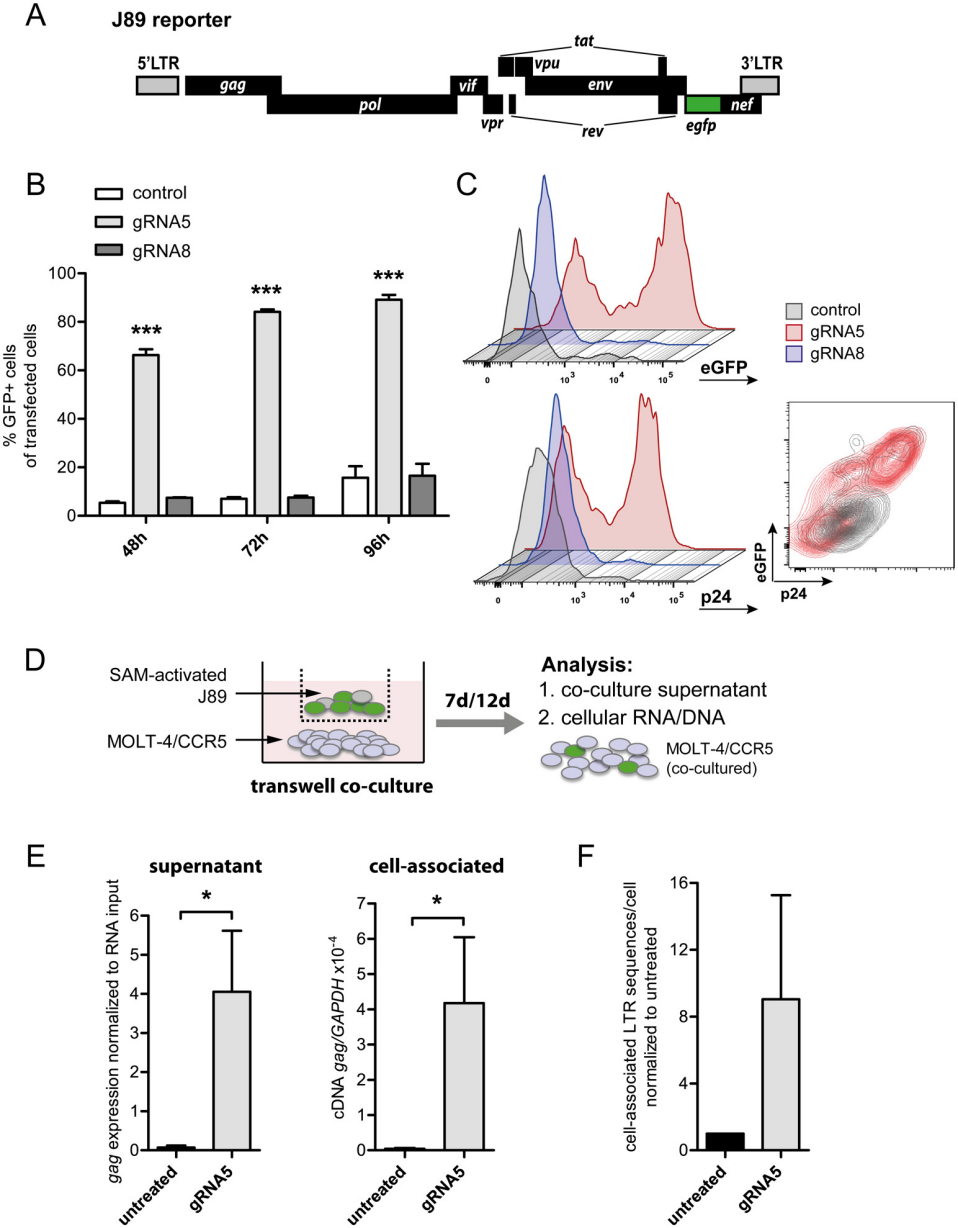
Induction of HIV replication using SAM in the J89 latency model. (**A**) Scheme of replication-competent HIV reporter virus in J89 clones. Transcriptional activation results in LTR-dependent eGFP expression and viral replication. (**B**) Components of the SAM system together with gRNA5, gRNA8 or no gRNA (control) were transiently expressed in J89 cells and proviral activation was determined by flow cytometry at 48, 72, and 96 h post transfection. Two-way ANOVA was used for statistical evaluation (in relation to control); *** signifies p<0.001. Shown are results of three independent experiments. (**C**) Number of J89 cells showing eGFP expression (upper panel) and cell-associated p24 expression (lower panel) at 72 h post transfection with SAM system components and gRNA5, gRNA8 or no gRNA (control). Inset shows flow cytometry contour plot of p24 and eGFP co-expression in control (grey) and SAM/gRNA5 transfected (red) cells. (**D**) To assess viral replication after SAM-mediated activation, J89 cells transfected with SAM components and gRNA5, were sorted for eGFP expression and co-cultured at 72 h post transfection with MOLT-4/CCR5 cells using a transwell system. (**E**) At 7 days post initiation of the co-culture, levels of *gag* RNA in co-culture supernatants and MOLT-4/CCR5 cells (cell-associated) were measured using ddPCR. Co-culture of non-transfected J89 cells with MOLT-4/CCR5 cells served as control (untreated). Mann-Whitney *U* test was used for statistical evaluation (in relation to untreated); * signifies p<0.05. (**F**) At 12 days post initiation of the co-culture, cell-associated LTR DNA content was determined in MOLT-4/CCR5 cells. Levels are shown as fold increase of LTR sequences per cell over untreated control. Shown are results of two independent experiments (E, F).

Several currently applied LRAs may only promote incomplete induction of latent provirus, failing to stimulate production of fully replication-competent viral progeny [8]. To test whether robust SAM-mediated activation of HIV LTR-driven transcription can also induce production and release of infectious viral particles in formerly latent cells, we employed the J89 model system [33]. As opposed to JLat6.3 and HIVis systems, Jurkat-derived clonal J89 cells carry a fully replication-competent HIV-derived provirus with an eGFP marker (Fig 4A). This reporter provirus is latent under non-induced culture conditions (<2% GFP positive cells) [33]. Consistent with our results described above, transient expression of SAM and gRNA5 resulted in strong induction of J89 cells, with 66% GFP positive cells after 48 h and up to 89% induced cells at 96 hours (Fig 4B). Induction was also accompanied by up-regulation of cell-associated p24 levels in formerly latent J89 cells (Fig 4C).

To test for viral particle production, we used a transwell culture system to co-culture SAM-activated, GFP-expressing J89 cells (72 h post transfection with SAM/gRNA5) with MOLT-4/CCR5 cells. As previously described, this transformed CD4^+^ T cell line served as a host to amplify any J89-derived infectious particles (Fig 4D) [8]. Co-culture of non-transfected J89 cells with MOLT-4/CCR5 cells served as control (untreated). After seven days of co-culture, *gag* RNA levels were measured by digital droplet PCR (ddPCR) in both co-culture supernatant and MOLT-4/CCR5 host cells. We detected significant levels of *gag* RNA in co-culture supernatant after seven days, indicating viral particle release from SAM-activated J89 cells (Fig 4E, S4 Fig). Consistent with this finding, we observed significant levels of cell-associated *gag* RNA in MOLT-4/CCR5 cells exposed to activated J89 (Fig 4E, S4 Fig). These results indicate that MOLT-4/CCR5 cells were infected by J89-derived viral particles induced through SAM-mediated activation. In support, we also detected levels of HIV-LTR-derived DNA in MOLT-4/CCR5 cells after twelve days of co-culture with activated J89 cells (Fig 4F, S4 Fig). Taken together, these data indicate that targeted activation using the SAM system can transform latent HIV provirus into actively replicating states.

### Functional effects of sequence variations in optimal target gRNA on SAM-mediated LTR activation

Having demonstrated that SAM components together with gRNA5 can specifically activate latent HIV genomes, our results prompt the possibility of using this activation system on latent reservoirs *in vivo*. Since LTR sequences in HIV isolates often diverge among and between viral subtypes, we therefore went on to ask how variations in optimal gRNA sequences affect SAM-mediated provirus activation.

**Fig 5:**
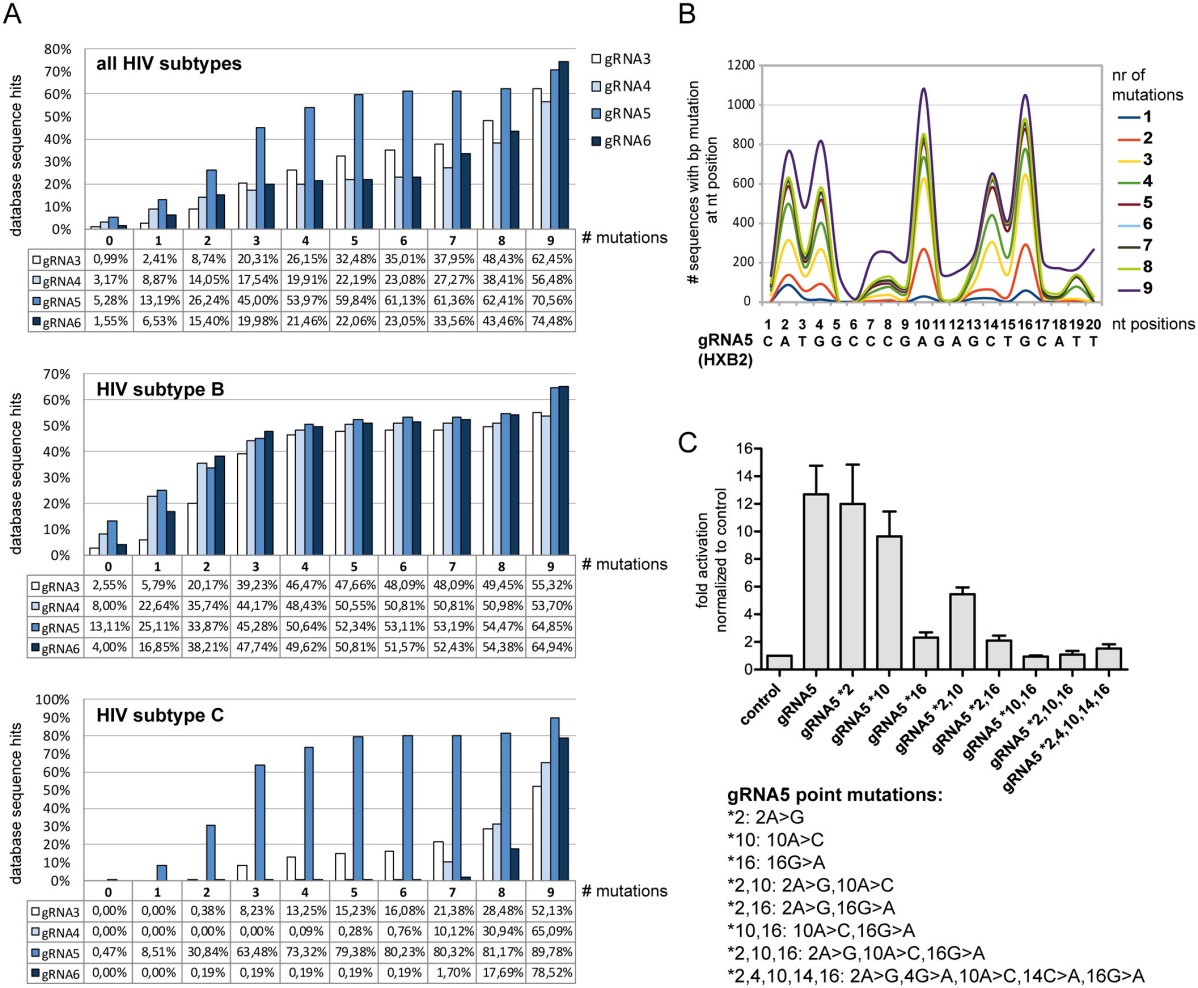
Conservation of optimal gRNAs and the effect of sequence variation on SAM-mediated LTR activation. (**A**) Sequence conservation of HXB2-derived gRNA3, -4, -5 and -6 based on the Los Alamos HIV Sequence Database considering all HIV subtypes (upper panel), subtype B only (middle panel) or subtype C only (lower panel). Percentages of sequence hits are shown, allowing for 0 to 9 point mutations in the indicated gRNA (see Materials and Methods for details). (**B**) Frequency distribution of gRNA5 mutations observed in database hits according to nucleotide (nt) position. Different colors indicate the number of mutations (1–9). (**C**) The effect of sequence variations in gRNA5 on SAM-mediated LTR activation was tested by transient expression of gRNA mutants (gRNA *X, where X is the mutated nucleotide position) with SAM system components in TZM-bl cells. Activation levels were measured at 48 h post transfection by luciferase assays performed on whole cell lysates. Activation is shown as fold increase (light units/transfected cell) over negative control (no gRNA expression). Shown are results of two independent experiments.

We first determined to what extent gRNA3, -4, -5 and -6 sequences are conserved within the optimal LTR target region based on the Los Alamos National Laboratory HIV sequence database (Fig 5A; www.hiv.lanl.gov). Among all HIV-1 subtypes, as well as subtype B or C alone, the gRNA5 sequence is the most conserved, present in 5% of all deposited sequences, and in 13% of subtype B or 0.5% of subtype C isolates. Allowing for point mutations within the 20 nt gRNAs, tolerating two mutations increases sequence conservation of gRNA5 to 26% for all subtypes, 34% for subtype B and 31% for subtype C. For all retrieved HIV sequences, our database searches revealed that gRNA3, -4 and -6 are much less conserved than gRNA5, with at least nine mutations required for a sequence match of 50% or more, as opposed to four mutations for gRNA5 (Fig 5A).

Focusing on gRNA5, we further examined the nucleotide positions that vary most frequently in this gRNA target sequence: variation primarily occurs at nucleotides two and four in the 5’ region, position 10 in the central region, and positions 14 and 16 in the seed region of the gRNA (Fig 5B). To test how mutations at these various positions affect SAM-mediated activation, we generated mutants of gRNA5. In these mutants nucleotides were substituted according to frequently occurring variations at the specific site (e.g. gRNA5 *2 indicates gRNA5 2A>G; see S5 Fig). We then co-expressed these gRNA5 mutants and SAM components in TZM-bl cells and assayed for luciferase reporter activity. Mutations at positions 2 or 10 in gRNA5 had little effect on SAM-mediated LTR activation (gRNA5 *2, gRNA5 *10; Fig 5C). Mismatches at both positions reduced activation by about 50% (i.e. gRNA5 *2,10). However, mutations within the seed region (e.g. gRNA5 *16) virtually abolished SAM-mediated LTR induction. This observation is in agreement with previously published data showing that sequence mismatches immediately upstream of the PAM site abolish binding of gRNAs to their respective targets [34].

## Discussion

Here, we present a proof of concept that CRISPR/Cas9-derived activation systems can be used to induce latent HIV genomes. In particular, using the SAM system we achieved strong and reproducible activation of latent proviruses, which importantly continued to the level of producing infectious particles. We identified an optimal target region for recruiting CRISPR/Cas9 activation systems to the HIV 5’LTR and demonstrate how sequence variations within this region affect system activity.

Our findings are supported by recent studies that show *in vitro* reversal of HIV latency using various engineered transcriptional activation systems based on CRISPR/Cas9 technology [35–38]. These studies focused on different effector modules, such as fusions of dCas9 to VP64 (dCas9-VP64), to VP64, p65 and Epstein-Barr virus R transactivator (Rta; dCas9-VPR), to histone acetyltransferase p300 (dCas9-p300) as well as the SAM and SunTag systems. Independently of the effector, all studies agree with our findings that CRISPR/Cas9-derived technologies can induce transcription from latent HIV LTR in all culture models tested so far. Furthermore, there is evident overlap in the target region identified for optimal recruitment of activator complexes to HIV 5’LTR. Our data and published reports strongly indicate that gRNAs complementary to sequence 164bp to 92bp upstream of the transcriptional start site lead to optimal targeting (S6 Fig). Certain differences exist concerning the sequence of the most efficient gRNA within this region, however these may be explained by variations in the reporter systems used to test gRNA effectiveness and by differences in activator modules. A comprehensive comparison between different CRISPR/Cas9-derived activator modules and single as well as multiplexed targeting gRNAs should in future further clarify this issue.

Notably, in the context of recent reports on CRISPR/Cas9-based activators for HIV latency reversal, our study focuses in particular on how viral gene expression correlates with LTR-targeted activation. Here we are the first to demonstrate that induction using CRISPR/Cas9-derived systems is complete, i.e. results in release of infectious particles, and thus transforms dormant proviruses into replicative viral states in a T-cell derived model. This key finding qualifies CRISPR/Cas9-based technologies for translational applications.

CRISPR/Cas9-derived approaches have a number of potentially significant advantages over currently used LRAs. They can be used for LTR-targeted induction of latent HIV genomes, reaching often much higher levels than reported for LRAs (see above; [35–38]). Their potential for site-specific activation determined by gRNA sequence choice contrasts with currently applied LRAs. These modulate global gene expression via cellular pathways, and harbor therefore considerable potential for undesired or toxic effects [39]. Notably, we provide evidence that targeting of CRISPR/Cas9-derived systems can result in activation up to the release of infectious particles. Such full activation is thought to be an essential prerequisite for purging latent reservoir cells through immune surveillance as part of current eradication strategies [1]. It is also required for *ex vivo* identification and effective quantification of the reservoir—an aspect of increasing interest and concern in view of tailoring suppressive therapy regimens. Furthermore, as CRISPR/Cas9-derived activators circumvent activation of cellular pathways, they hence likely reverse latency independently of cellular identities and activity states [37]. Since current findings point towards an increasingly heterogeneous reservoir, such activators therefore have tremendous potential to revert latency across the full spectrum of dormant HIV-infected cells [3]. Clearly, this may also hold true for TALE-based HIV activating strategies [16,18]. However, in direct comparison, the ease with which CRISPR/Cas9-derived systems can be adapted to new target sequences may confer a considerable advantage.

In the future, current findings will need to be expanded to primary and patient-derived HIV-infected cells. These studies will allow to address potential toxicity of CRISPR/Cas9-based activator systems and mechanistic details of transcriptional induction, such as for example protein levels bound to DNA and epigenetic changes at the LTR promoter. In this context, it will be critical to develop effective and safe vectors for delivery into primary cells. While the size of activator modules currently prevents packaging into clinically preferred adeno-associated virus (AAV) vectors [40], rapid technical advances in the field of CRISPR/Cas9-technologies will likely soon circumvent this drawback. Sequential lentiviral delivery of activator complexes into clonal cell lines has already been employed [35,38]. However, such applications are far from reaching clinical translation. Here, we opted for transient transfection of activator complexes to provide a proof of principle.

Our analysis of sequence conservation among targeting gRNAs within the HIV 5’LTR touches on a second aspect relevant for translating CRISPR/Cas9-derived activators to clinical applications. Our data indicate that 5’ mutations in gRNAs are likely to be tolerated, while variations within the seed region abolish directed targeting, as previously reported [34]. Since latent reservoirs harbor a variety of viral mutants, successful activation through CRISPR/Cas9-derived technologies will need to take into account sequence variations [41]. To bypass any escape, delivery of multiple guide RNAs tailored towards these variations could be a successful approach. Indeed, our findings indicate that expressing multiple gRNAs does not interfere, but rather can potentiate CRISPR/Cas9-mediated activation.

In conclusion, much effort has been directed towards finding specific and potent LRAs to eliminate HIV latent reservoirs. Here, we demonstrate that activation systems based on CRISPR/Cas9-technology could be potent tools for this purpose. These tools are waiting to be exploited to complement future latency reversing strategies *ex vivo* or potentially in the patient.

## Materials and Methods

### Plasmid construction

Expression plasmids for components of the SunTag and SAM system were obtained from Addgene: pHRdSV40-dCas9-10xGCN4_v4-P2A-BFP (Addgene #60903, SunTag), pHRdSV40-scFv-GCN4-sfGFP-VP64-GB1-NLS (Addgene #60904, SunTag), dCas9-VP64_GFP (Addgene #61422, SAM), MS2-P65-HSF1_GFP (Addgene #61423, SAM). For expression of SAM components in JLat6.3 and J89 cells, the T2A-GFP sequence was replaced by the T2A-BFP sequence at the *Nhe*1/*Eco*R1 restriction sites (MS2-P65-HSF1_BFP). The gRNA sequences were derived from HXB2 (TZM-bl, JLat6.3, J89 cells) and NL4-3 (HIVisB2 cells) viral genomes. For expression, gRNA sequences were cloned as oligonucleotides (Sigma) into pSUPER (Oligoengine; SunTag system; H1 promoter-dependent expression) or sgRNA(MS2) cloning backbones (Addgene #61424; SAM system; U6 promoter-dependent expression). To compare effects of complete versus incomplete SAM system expression (see S1 Fig), the following expression constructs were used: MS2-P65-HSF1_BFP, lenti_sgRNA (MS2)_SFFV_Venus (based on Addgene #61427 with insertion of SFFV_Venus sequences at the *Bam*H1/*Eco*R1 restriction sites) and dCas9-VP64_mCherry (based on Addgene #61422 with replacement of the T2A-GFP sequence by the T2A-mCherry sequence at the *Nhe1*/*EcoR1* restriction sites). The proviral construct pNLT2-HIVis was generated by replacing the *Blp*I-*Xho*I fragment of construct pNLT2ΔenvPuro [42] with the coding sequence for BFP followed by the SFFV promoter and coding sequence for Venus-fluorescent protein (Fig 3A).

### Cell culture

The following reagents were obtained through the NIH AIDS Reagent Program, Division of AIDS, NIAID, NIH: TZM-bl from Dr. John C. Kappes, Dr. Xiaoyun Wu and Tranzyme Inc. [27]; J-Lat Full Length Clone 6.3 from Dr. Eric Verdin [28]; MOLT-4/CCR5 from Dr. Masanori Baba, Dr. Hiroshi Miyake, and Dr. Yuji Iizawa. J89 cells were a kind gift from Dr. David N. Levy (New York University) [33]. HEK293 (ATCC catalogue #CRL-1573) and TZM-bl cells were cultured in Dulbecco’s modified Eagle medium (DMEM, Biochrom) supplemented with 10% fetal calf serum (Biochrom), 4 mM L-glutamine (Biomol), 50 U/ml penicillin and streptomycin (PenStrep, Biochrom) at 37°C and 5% CO_2_. Jurkat (ATCC catalogue #TIB-152), JLat6.3, HIVisB2 and J89 cells were maintained in RPMI 1640 (Lonza) supplemented with 10% FCS (PAN, Biotech), 4 mM L-glutamine, 50 U/ml penicillin and streptomycin (PenStrep; Biochrom) at 37°C and 5% CO_2_. Media for MOLT-4/CCR5 was additionally supplemented with 1 mg/ml G418 (Gibco Life Technologies).

### HIVis infection and generation of HIVisB2 cell line

Pseudotyped HIVis lentiviral particles were produced as previously described [43]. In short, HEK293 cells were transiently transfected with proviral pNLT2-HIVis plasmid and pCMV-VSV-G [44] using transfection reagent TransIT-LT1 (Mirus Bio) according to the manufacturer’s protocol. Viral supernatants were collected 72 h post transfection. To generate Jurkat-HIVis cells, Jurkat cells were infected with pseudotyped pNLT2-HIVis (MOI 0.1) in the presence of 5 μg/ml protamine sulfate (Sigma-Aldrich) and spinoculated at 300 x g for 10 min at ambient temperature. After spinoculation cells were cultivated at 37°C and 5% CO2 for 5 h and subsequently medium was changed. Jurkat-HIVis cells were sorted for Venus-positive cells on a fluorescent activated cell sorting (FACS) Aria system (BD Biosciences) 4 weeks after infection. Single cell clones were generated via repeated limiting dilution in 96-well plates to identify the HIVisB2 clone. Proviral integration site was determined by HiLo-PCR as described before [43].

### Transient transfection assays, firefly-luciferase reporter assay and treatment with LRAs

For transient expression of SunTag or SAM system components, TZM-bl cells were seeded at 2 x 10^5^ cells/ml and JLat6.3, HIVisB2, or J89 cells at 5 x 10^5^ cells/ml in 6-well plates 24 h prior to transfection. Transient transfection of TZM-bl cells was performed with Lipofectamine 2000 (Thermo Fisher Scientific) according to the manufacturer’s protocol. JLat6.3, HIVisB2 and J89 cells were transfected using TransIT-Jurkat (Mirus Bio) according to the manufacturer’s protocol. Cells were co-transfected in triplicate with the following amounts of plasmid/6-well: 1) SunTag system: 0.9 μg pH1-gRNA1-9, 1.2 μg pHRdSV40-dCas9-10xGCN4_v4-P2A-BFP and 1.9 μg pHRdSV40-scFv-GCN4-sfGFP-VP64-GB1-NLS; 2) SAM system: 0.9 μg sgRNA(MS2)-gRNA1-9, 1.3 μg dCas9-VP64_GFP and 1.8 μg MS2-P65-HSF1_GFP. For luciferase reporter assays, whole cell lysates were prepared 48 h post transfection and treated using Luciferase Reporter Assay System (Promega) according to the manufacturer’s protocol. Measurements were performed using a Centro LB 960 luminometer (Berthold Technologies) and MikroWin2000 software (Mikrotek). For treatment with LRAs, cells were seeded at 1 x 10^6^ cells/ml and treated with the following concentrations of agents unless otherwise stated: 40 nM Romidepsin (MedChem Express), 335 nM Vorinostat (Sigma Aldrich), 300 nM Prostratin (Sigma Aldrich), 50 ng/ml phorbol 12-myristate 13-acetate (PMA; Sigma Aldrich) or 1 μM Ionomycin (Sigma-Aldrich).

### AlamarBlue cell viability assay

For cell viability assessment, 100 μl of treated cell cultures were plated in triplicate in 96-well plates and 20 μl AlamarBlue reagent (AbD Serotec) was added. Plates were incubated at 37°C and 5% CO_2_ for 2 h and absorbance was measured at 570/600 nm using a VERSAmax microplate reader (Molecular Devices). Viable cells reduce the active ingredient of AlamarBlue reagent (resazurin) to resorufin, which absorbs at 570/600 nm.

### Transwell co-culture assay

J89 cells were transfected with SAM system components and enriched for GFP-positive cells 72 h post transfection using a FACS Aria Fusion (BD Biosciences). Sorted J89 and MOLT-4/CCR5 recipient cells were co-cultured in 24-well Transwells (PET membrane, 0.4 μm pore size, Corning Costar) at a ratio of 1:20 (J89 upper well, MOLT-4/CCR5 lower well). At 7 days post co-culture, supernatant was taken from the transwell system for RNA isolation. At the same time point, 50% of MOLT-4/CCR5 cells were taken from the co-culture for isolation of cell-associated RNA. At 12 days post co-culture, the remaining MOLT-4/CCR5 cells were isolated from the co-culture for genomic DNA isolation.

### Flow cytometry, p24 staining and immunoblot analysis

Transfected cells were washed twice in PBS and analyzed using a Canto II flow cytometer (BD Biosciences). J89 cells were fixed in 2% paraformaldehyde (Sigma Aldrich) prior to analysis. Data was analyzed using FlowJo vX.0.7 software (Tree Star). To account for transfection efficiency, JLat6.3 and J89 cells were gated on BFP expression and HIVisB2 cells were gated on GFP expression. For intracellular p24 staining, HIVisB2 cells were enriched for GFP-expressing transfected cells using a FACS Aria system (BD Biosciences). Transfected J89 and HIVisB2 cells were washed twice with PBS and fixed with 4% paraformaldehyde for 20 min in the dark. Cells were then washed with Perm/Wash buffer (BD Biosciences) and incubated with 250 μl Cytofix/Cytoperm solution (BD Biosciences) for 30 min at 4°C in the dark. Cells were washed again and stained with 2 μl of anti-HIV-1 core antigen clone KC57-RD1 (Beckman Coulter) in 50 μl of Perm/Wash buffer for 60 min at 4°C in the dark. Cells were washed and subsequently analyzed using a Canto II flow cytometer (BD Biosciences). For immunoblot analysis, cell pellets were lysed in E1A lysis buffer (150 mM NaCl, 50 mM HEPES, pH 7.0, 0.1% Nonidet P-40, 5 μg/ml leupeptin, 5 μg/ml aprotinin, 5 μg/ml pepstatin A, 125 μg/ml pefabloc (all protease inhibitors from Biomol)) on ice for 30 min. Protein samples were resolved by electrophoresis on a 15% SDS polyacrylamide gel and blotted onto nitrocellulose membrane. HIV-1 Gag and β-Tubulin were detected with anti-Gag (clone CHE-CAP, diluted 1:10,000, Davids Biotechnologie) and anti-α-Tubulin (clone B-5-1-2, diluted 1:5,000, Sigma-Aldrich) and visualized with IRDye-coupled secondary antibodies (diluted 1:10,000, LI-COR Biosciences) on a LI-COR Odyssey Imaging System (LI-COR Biosciences).

### qRT-PCR and ddPCR analysis

For qRT-PCR, total cellular RNA was isolated using peqGOLD TriFast (peqlab) according to the manufacturer’s protocol. DNase treatment was performed using RQ1 DNase (Promega) and DNase-treated RNA samples were reverse transcribed with M-MLV reverse transcriptase (Promega) using an oligo-dT primer mix. Quantitative real-time PCR (qRT-PCR) was carried out using Platinum Quantitative PCR SuperMix-UDG (Life Technologies) on a 7500 Fast Real-Time PCR System (Applied Biosystems). The following primers were used for qRT-PCR:

tat-F (GGCATCTCCTATGGCAGGAA), tat-R (TGCTTTGATAGAGAAACTTGATGAGTCT), tat-probe (5'(FAM)-ACAGCGACGAAGAGCTCATCAGAACAGT-(TAMRA)3'),

gag-F (ATCAATGAGGAAGCTGCAGAA), gag-R (GATAGGTGGATTATGTGTCAT), gag-probe (5'-(FAM)-ATTGCACCAGGCCAGATGAGAGAA-(TAMRA)-3'),

gapdh-F (GTCATCAATGGAAATCCCATCA), gapdh-R (TGGTTCACACCCATGACGAA), gapdh-probe (5'-(FAM)-TCTTCCAGGAGCGAGATCCCTC-(TAMRA)-3').

The amplification profile involved initial denaturation at 95°C for 3 min and 40 cycles of denaturation at 95°C for 15 sec and hybridization and elongation at 60°C for 30 sec. All samples were assayed in quadruplicate wells. For ddPCR analysis, RNA from supernatant and total cellular RNA was isolated using RNAzol (Sigma-Aldrich) according to the manufacturer’s protocol. Genomic DNA was isolated using QIAamp DNA micro Kit (Quiagen). Nucleic acid quantities were determined by NanoDrop (Thermo Fisher Scientific). ddPCR for HIV *gag* RNA was carried out using the QX200 platform from BioRad. cDNA samples were prepared as described above. For the quantification of HIV *gag* RNA, primers and probe as indicated for qRT-PCR were used (quencher BHQ1 instead of TAMRA). For cellular HIV *gag* RNA expression analysis, *gapdh* expression was determined as a control using the PrimePCR ddPCR Expression Probe Assay: GAPDH (Human, HEX; BioRad). All samples were assayed in duplicate wells. The mastermix/well included the following: 2 x ddPCR Supermix for Probes (noUTP) (BioRad) 1:2, gag-F (1 μM), gag-R (1 μM), gag-probe (250 nM), template cDNA 0.5 μl, filled up to 20 μl total volume with RNase free water. When measured target concentrations were very low, template input was increased to 5 μl and runs were repeated. For *gapdh* expression analysis, separate duplicate wells/sample were prepared, the template cDNA was diluted 1:10, and 1 μl of the GAPDH Expression Probe Assay was added to the 20 μl reaction (900 nM primers, 250 nM probe). “No-template” controls (NTC) containing water instead of cDNA were included in duplicates for all assays. Subsequently, this mastermix was used for oil droplet generation using a QX200 Droplet Generator, according to the manufacturer’s instructions. The droplets were transferred to 96-well twin.tec PCR Plates (Eppendorf), the plates were heat-sealed with tin foil and placed in a thermal cycler with a 105°C heated lid (Analytik Jena). Thermal cycling was carried out using the following parameters: 1 x (95°C 10 min), 40 x (94°C 30 sec– 55°C 1 min), 1 x (98°C 10 min), 4°C (hold), with a 2°C/sec ramping rate. After thermal cycling, plates were immediately placed into the QX200 ddPCR Plate Reader, and droplets were analyzed using QuantaSoft software, version 1.7.4. Following the run, amplitude and cluster data were exported, and fluorescence thresholds for positive-negative event discrimination and target concentrations in the ddPCR runs were determined using the function ‘ddPCRquant’ in R Studio (version 0.99, RStudio Inc.) [45]. For cell-associated HIV DNA analysis, primers and a 6-FAM-labeled fluorescent probe binding in a highly conserved region of the LTR in the HIV genome were used (‘Generic HIV DNA Cell’, Biocentric) [46]. To quantify HIV genomes/cell, the concentration of the single copy gene *rpp30* was determined in parallel (PrimePCR ddPCR Copy Number Assay RPP30, HEX, BioRad). The mastermix/well included the following: 2 x ddPCR Supermix for Probes (noUTP) 10 μl, HIV-LTR forward and reverse primers as well as the HIV-LTR probe (400 nM each), RPP30 Copy Number Assay (900 nM primers/250 nM probe) and 5 μl template DNA, filled up to 20 μl total volume with RNase free water. Droplet generation and reading, as well as data analysis was carried out as described for RNA measurements, the thermal cycling protocol was as follows: 1 x (95°C 10 min), 40 x (94°C 30 sec– 56.2°C 1 min), 1 x (98°C 10 min), 4°C (hold), with a 2°C/sec ramping rate.

### Bioinformatics analysis

Bioinformatics analysis was based on the Los Alamos National Laboratory HIV sequence database (www.hiv.lanl.gov). All HIV-1-sequences within the database were filtered for sequences containing the genomic region from base position 241 to 348 (HXB2 annotation), resulting in a set of 3033 sequences. Using this set, all sequences with a specific Hamming distance to gRNA3, -4, -5 or -6 were counted, and the exact positions of the matching errors were estimated.

To count sequences, for Hamming distance *d*_*H*_ and gRNA-sequence *s*_*gRNA*_ with length *l*_*gRNA*_, every sequence *s*_*db*_ of the HIV-database with length *l*_*db*_ was split into k-mers of length *k* = *l*_*gRNA*_, resulting in *n* = *l*_*db*_ − *l*_*gRNA*_ + 1 k-mers. If one k-mer with Hamming distance *d*_*H*_ between k-mer and *s*_*gRNA*_ existed, the sequence count was increased by 1. For each of the *n* k-mers with Hamming distance *d*_*H*_ between k-mer and *s*_*gRNA*_ the exact positions of the matching errors were determined. Finally, a multiple sequence alignment (MSA) over all k-mers of the HIV-database with Hamming distance *d*_*H*_ between the k-mer and *s*_*gRNA*_ was generated with Clustal Omega [47]. Afterwards the MSA was used to generate a sequence logo using Weblogo 2.8.2 [48].

### Statistical analysis

Statistical analysis was performed using Prism version 5.03 software (Graph Pad). The statistical significance was assessed by one-way or two-way analysis of variance (ANOVA) followed by a Bonferroni’s Multiple Comparison Test or Mann-Whitney *U* test. A result of p<0.05 was considered to be statistically significant.

## Supporting Information

S1 Fig Comparison of SAM-mediated activation to single dCas9-VP64- or MS2p65HSF1-mediated effects. The effect of gRNA5-targeted SAM system (dCas9-VP64 + MS2p65HSF1 + gRNA5) on LTR activation was compared to gRNA5-targeted expression of Cas9-VP64 or MS2p65HSF1 alone in transient transfection assays in TZM-bl cells carrying a luciferase reporter under HIV LTR control. Activation levels were measured 48 h post transfection using a luciferase assay on whole cell lysates. Activation is shown as fold increase (light units/transfected cell) over the negative control (no gRNA expression). Shown are results of two independent experiments.
(PDF)

S2 Fig Treatment of JLat6.3 and HIVisB2 with different concentrations of latency reversing agents. JLat6.3 and HIVisB2 cells were exposed to different concentrations of latency reversing agents (LRAs). Arrows indicate clinically relevant LRA concentrations as reported previously [30](see also Figs 2C and 3C). Proviral activation levels in (A) JLat6.3 and (B) HIVisB2 cells were measured by flow cytometry and cell viability was determined by AlamarBlue cell viability assay. Values for AlamarBlue viability assay were normalized to those of untreated cells. Shown are results of two independent experiments.
(PDF)

S3 Fig Characterization of HIVis latency reporter in Jurkat cells and clonal HIVisB2 cells. (**A**) Jurkat cells were transduced with VSV-G pseudotyped HIVis reporter, enriched for BFP negative/Venus positive cells by FACS and exposed to 670 nM Ionomycin and 80 nM PMA for 6 h or left untreated. Cell-associated *tat* RNA levels in Venus expressing Jurkat-HIVis cells were measured by qRT-PCR over a period of 7 days after stimulation and normalized to *gapdh* RNA levels. (**B**) Levels of HIV Gag and cellular Tubulin were measured via immunoblots (6 μg protein per sample). (**C**) Clonal HIVisB2 cells show LTR-dependent BFP expression after stimulation with 10 nM PMA and 1 μM Ionomycin for 24 hours.
(PDF)

S4 Fig Analysis of raw ddPCR data by ‘ddPCRquant’ in R Studio. After baseline alignment, fluorescent threshold values for each run were calculated based on data from no-template control (NTC) wells using extreme value theory. Representative dot plots (NTC and baseline, MOLT-4/CCR5 + untreated control J89 co-culture, MOLT-4/CCR5 + SAM/gRNA5 transfected J89 co-culture) are shown for (**A**) co-culture supernatant *gag* cDNA, (**B**) co-cultured MOLT-4/CCR5 cell-associated *gag* cDNA and (**C**) MOLT-4/CCR5 cell-associated HIV-1 LTR DNA assays. See Materials and Methods for details on the experimental setup.
(PDF)

S5 Fig Sequence logos showing residue conservation of HXB2-derived gRNA5. The Los Alamos HIV Sequence Database (all HIV subtypes) was used for conservation studies. Sequences are shown for 0 to 9 allowed point mutations.
(PDF)

S6 Fig Optimal gRNAs for recruitment of CRISPR/Cas9-derived activator systems to the HIV 5’ LTR. Scheme of HIV 5’LTR showing localization of gRNAs (arrows) that have been reported as most effective for recruitment of different CRISPR/Cas9-derived activator systems in various independent studies (see table). Red arrows indicate localization of gRNAs3-6, which were found in present study to define an optimal target region based on SAM-mediated induction of TZM-bl reporter cells.
(PDF)
