# Supplementary figures and images for: Targeted HIV-1 Latency Reversal Using CRISPR/Cas9-Derived Transcriptional Activator Systems
Source: PLoS One. 2016 Jun 24;11(6):e0158294. doi: 10.1371/journal.pone.0158294 (PMC4920395; doi:10.1371/journal.pone.0158294)

Figure S1  
Bialek et al.

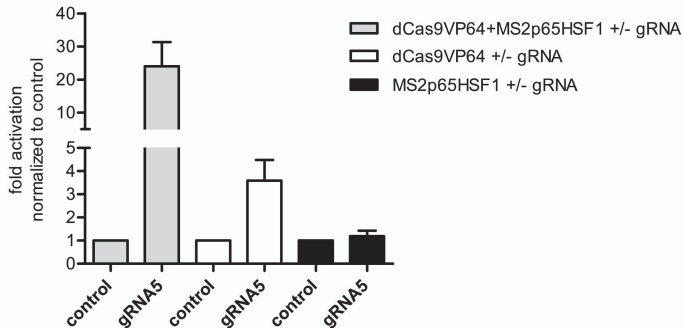

Supplement: S1 Fig — The effect of gRNA5-targeted SAM system (dCas9-VP64 + MS2p65HSF1 + gRNA5) on LTR activation was compared to gRNA5-targeted expression of Cas9-VP64 or MS2p65HSF1 alone in transient transfection assays in TZM-bl cells carrying a luciferase reporter under HIV LTR control. Activation levels were measured 48 h post transfection using a luciferase assay on whole cell lysates. Activation is shown as fold increase (light units/transfected cell) over the negative control (no gRNA expression). Shown are results of two independent experiments. (PDF) [file pone.0158294.s001.pdf]

Figure S2  
Bialek et al.

A

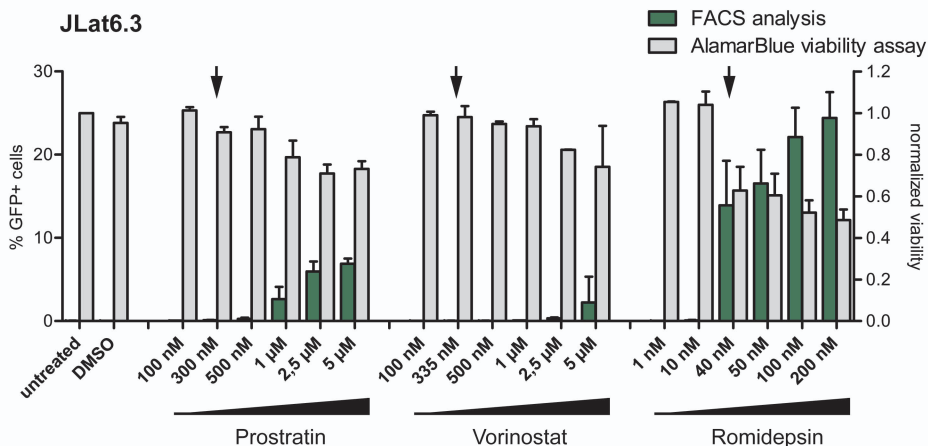

B

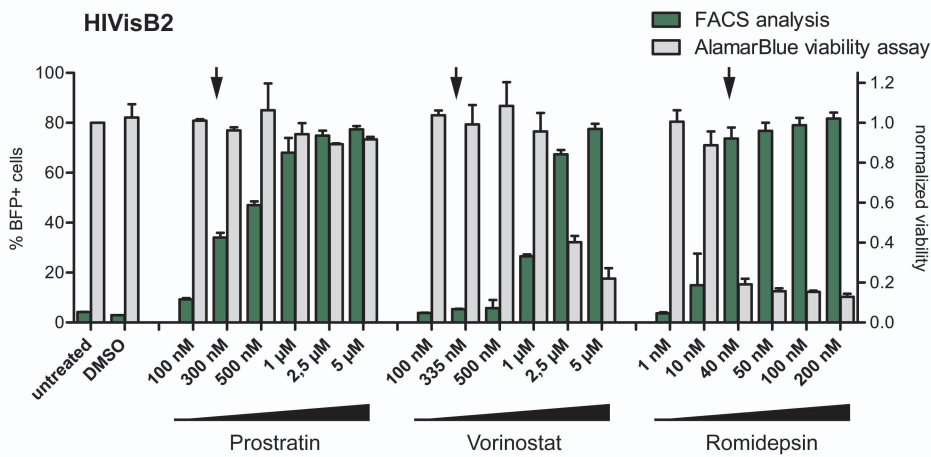

Supplement: S2 Fig — JLat6.3 and HIVisB2 cells were exposed to different concentrations of latency reversing agents (LRAs). Arrows indicate clinically relevant LRA concentrations as reported previously [30](see also Figs 2C and 3C). Proviral activation levels in (A) JLat6.3 and (B) HIVisB2 cells were measured by flow cytometry and cell viability was determined by AlamarBlue cell viability assay. Values for AlamarBlue viability assay were normalized to those of untreated cells. Shown are results of two independent experiments. (PDF) [file pone.0158294.s002.pdf]

Figure S3  
Bialek et al.

A

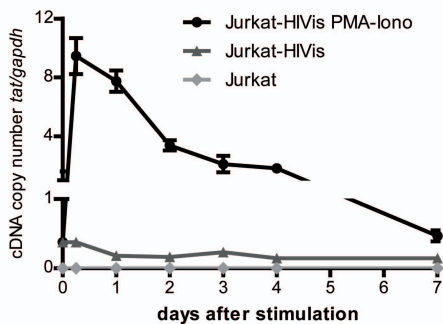

B

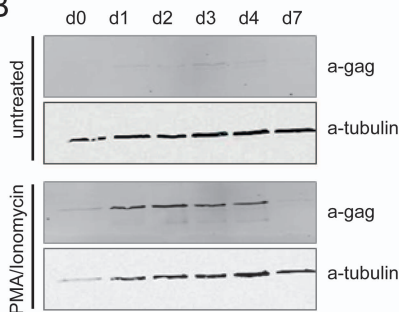

C

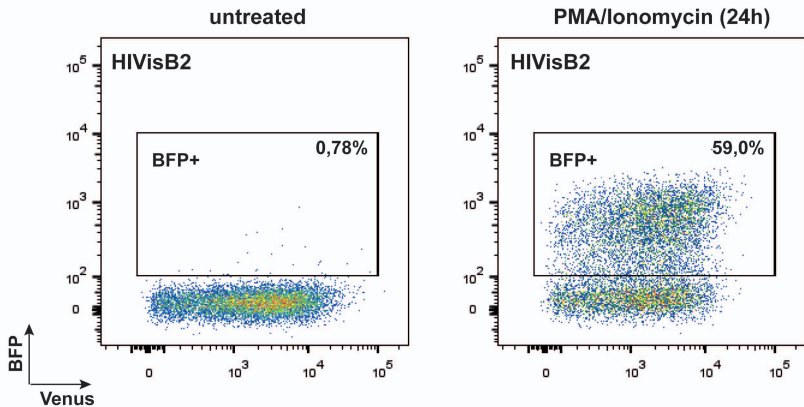

Supplement: S3 Fig — (A) Jurkat cells were transduced with VSV-G pseudotyped HIVis reporter, enriched for BFP negative/Venus positive cells by FACS and exposed to 670 nM Ionomycin and 80 nM PMA for 6 h or left untreated. Cell-associated tat RNA levels in Venus expressing Jurkat-HIVis cells were measured by qRT-PCR over a period of 7 days after stimulation and normalized to gapdh RNA levels. (B) Levels of HIV Gag and cellular Tubulin were measured via immunoblots (6 μg protein per sample). (C) Clonal HIVisB2 cells show LTR-dependent BFP expression after stimulation with 10 nM PMA and 1 μM Ionomycin for 24 hours. (PDF) [file pone.0158294.s003.pdf]

no-template control (NTC)

untreated

SAM/gRNA5

A

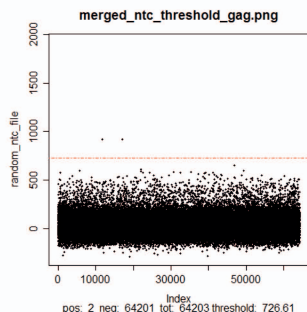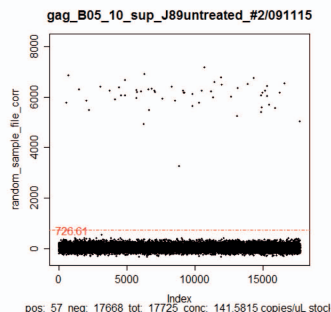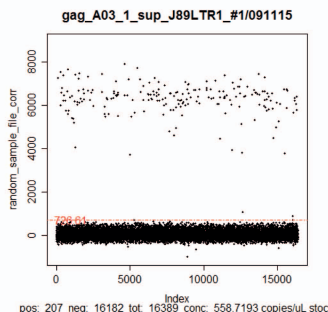

B

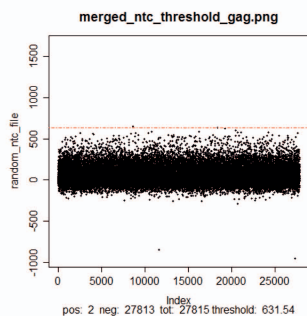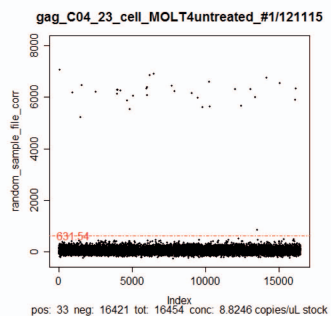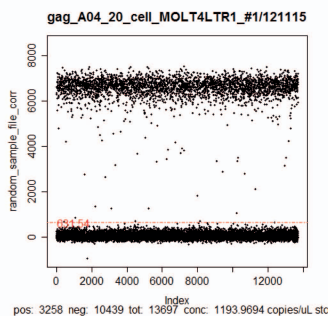

C

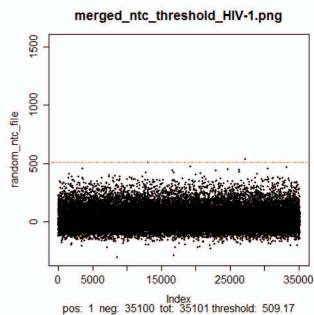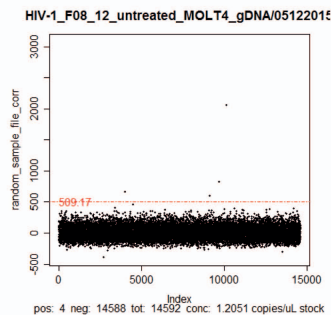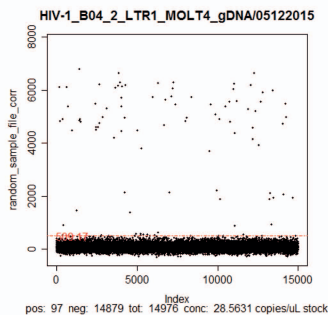

Supplement: S4 Fig — After baseline alignment, fluorescent threshold values for each run were calculated based on data from no-template control (NTC) wells using extreme value theory. Representative dot plots (NTC and baseline, MOLT-4/CCR5 + untreated control J89 co-culture, MOLT-4/CCR5 + SAM/gRNA5 transfected J89 co-culture) are shown for (A) co-culture supernatant gag cDNA, (B) co-cultured MOLT-4/CCR5 cell-associated gag cDNA and (C) MOLT-4/CCR5 cell-associated HIV-1 LTR DNA assays. See Materials and Methods for details on the experimental setup. (PDF) [file pone.0158294.s004.pdf]

Figure S5  
Bialek et al.

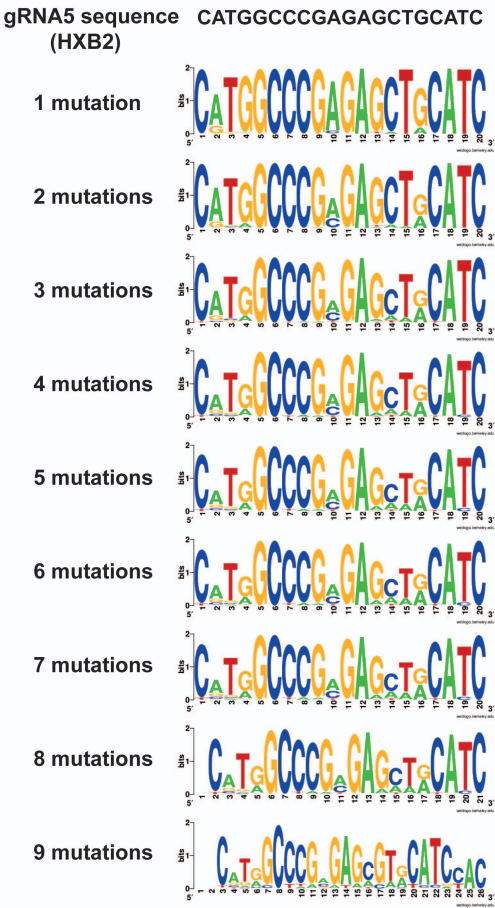

Supplement: S5 Fig — The Los Alamos HIV Sequence Database (all HIV subtypes) was used for conservation studies. Sequences are shown for 0 to 9 allowed point mutations. (PDF) [file pone.0158294.s005.pdf]
