# Supplementary material for: Targeted HIV-1 Latency Reversal Using CRISPR/Cas9-Derived Transcriptional Activator Systems
Source: PLoS One. 2016 Jun 24;11(6):e0158294. doi: 10.1371/journal.pone.0158294 (PMC4920395; doi:10.1371/journal.pone.0158294)
Supplement: S6 Fig — Scheme of HIV 5’LTR showing localization of gRNAs (arrows) that have been reported as most effective for recruitment of different CRISPR/Cas9-derived activator systems in various independent studies (see table). Red arrows indicate localization of gRNAs3-6, which were found in present study to define an optimal target region based on SAM-mediated induction of TZM-bl reporter cells. (PDF) [file pone.0158294.s006.pdf]

Figure S6

Bialek et al.

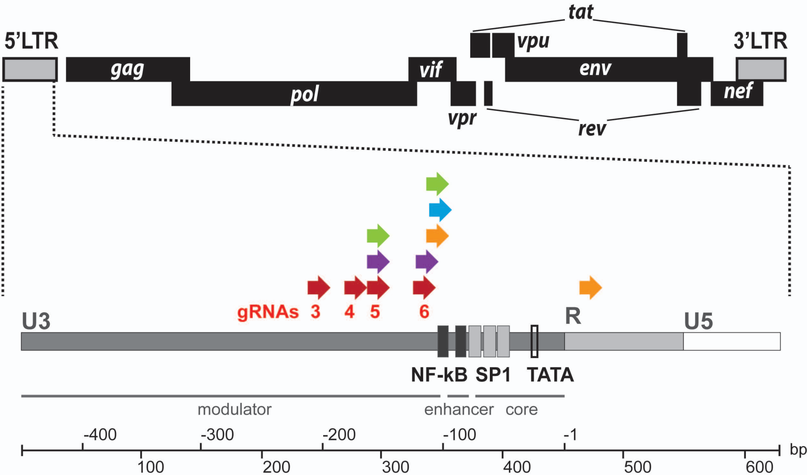

	gRNAs for activation	activator system	publication
green arrow	LTR-L, LTR-O	SAM	Zhang et al., 2015 (35)
blue arrow	sg362F	dCas9-VP64/VPR, SAM	Saayman et al., 2015 (37)
orange arrow	sgRNA 4, sgRNA 6	SAM, dCas9-VP64, dCas9-p300	Limsirichai et al., 2015 (38)
purple arrow	sgRNA 4, sgRNA 5	SunTag, dCas9-VP64	Ji et al., 2016 (36)
